# Designing and developing an app to perform Hofstee cut-off calculations

**DOI:** 10.12688/f1000research.53096.1

**Published:** 2021-06-07

**Authors:** Ken Masters, Nadia Al-Wardy

**Affiliations:** 1 Medical Education and Informatics, Sultan Qaboos University, Al-Khoud, 0123, Oman; 2 Biochemistry, Sultan Qaboos University, Al-Khoud, 0123, Oman

**Keywords:** Hofstee, Angoff, Assessment, Standard setting, Android, MARS

## Abstract

Determining a Hofstee cut-off point in medical education student assessment is problematic: traditional methods can be time-consuming, inaccurate, and inflexible.  To counter this, we developed a simple Android app that receives raw, unsorted student assessment data in .csv format, allows for multiple judges’ inputs, mean or median inputs, calculates the Hofstee cut-off mathematically, and outputs the results with other guiding information. The app contains a detailed description of its functionality.

## Introduction

### Determining the pass/fail score in assessments

In medical education assessment, determining the student pass/fail mark is a contentious issue. ^1^ A range of methods can be used to determine this point and are covered in several other papers. ^2^^–^^4^ In summary, however, most methods fall into three categories: norm-referenced (determined by the performance of the student group), criterion-referenced (pre-determined as an absolute cut-off point) and compromise methods (a compromise between the previous two methods is found). ^4^

### Hofstee method

The Hofstee Method ^4^^–^^6^ is a compromise method that follows four steps, and uses four variables or parameters (explained in more detail below) to determine the cut-off point.

*Step 1: Evaluation by judges*

In Step 1, judges who are qualified to assess the test make an independent judgement about the values of the following four parameters:

• c _min_: The minimum cut-off score (i.e. the score that the judge feels would be the lowest possible score that would be considered as a pass/fail score).
• c _max_: The maximum cut-off score (i.e. the score that the judge feels would be the highest possible score that would be considered as a pass/fail score).
• f _min_: The lowest percentage of students that the judge feels should fail this test.
• f _max_: The highest percentage of students that the judge feels should fail this test.

The four parameters are often indicated with different abbreviations; in this paper, we use c _min_, c _max_, f _min_ and f _max_ as is used elsewhere. ^4^ We should further note that judges will generally use the *Angoff* or similar method to determine these. ^4^

*Step 2: Determining the arithmetic means*

Based upon the independent judgements, the arithmetic mean of each parameter is calculated. (Some researchers, e.g. Norcini ^2^, have suggested that medians may also be used).

*Step 3: Plot on a graph*

**Figure 1.**
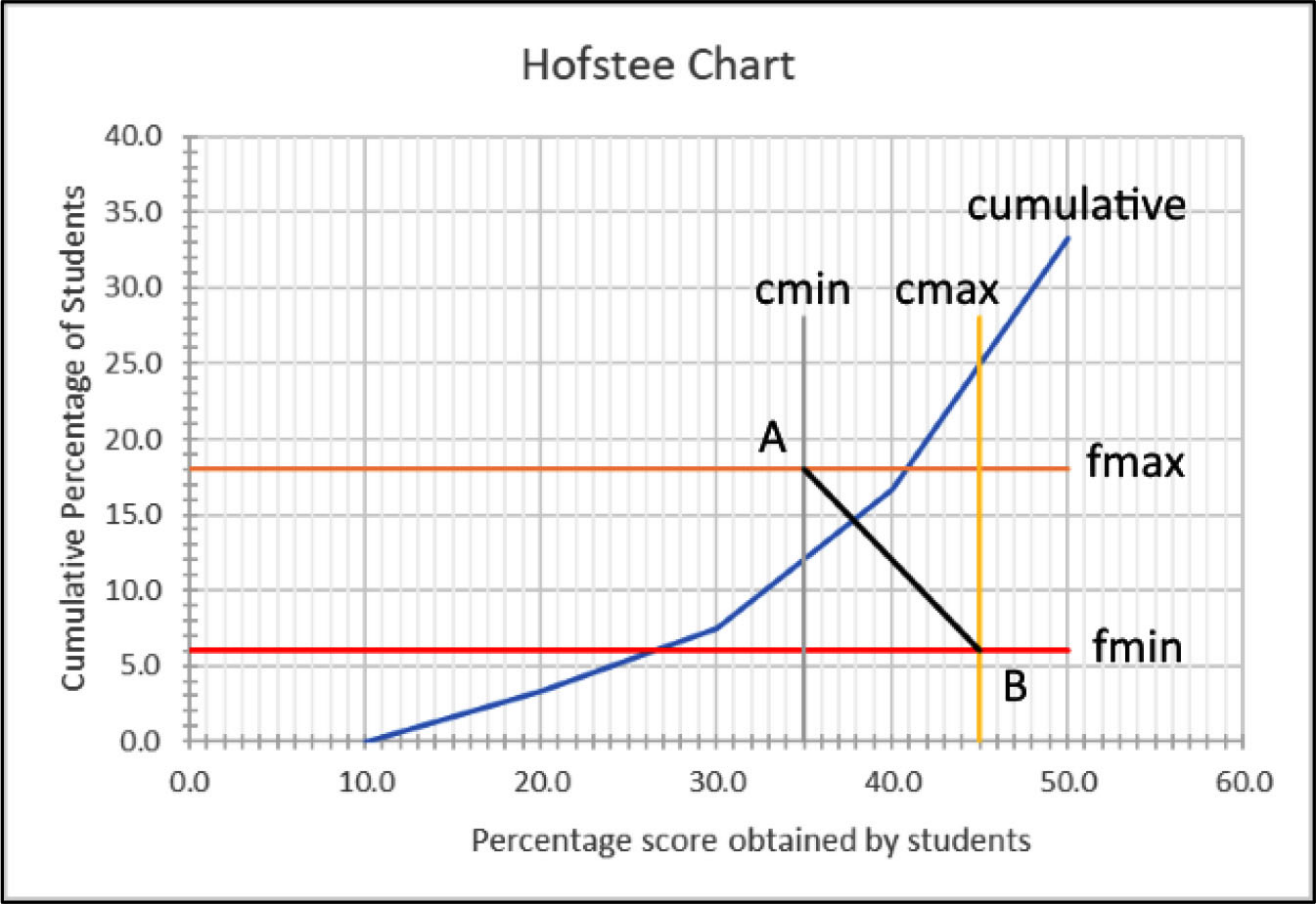
Hofstee chart showing cumulative scores, where c _min_ (minimum cut-off score) = 35, c _max_ (maximum cut-off score) = 45, f _min_ (the lowest percentage of students that the judge feels should fail) = 6, and f _max_ (the highest percentage of students that the judge feels should fail) = 18.

After the test has been administered to the students, a graph ( Figure 1) is then drawn, plotting the cumulative percentage of students against the scores obtained, and the four parameters.

*Step 4: Determining cut-off*

The pass/fail cut-off point is then determined by drawing line *AB* and finding the intersect with the cumulative line. In the Figure 1 example, the cut-off is determined to be slightly less than 38%.

### Practical problems with using the Hofstee method

Apart from the fact that any cut-off method can be debated, there are practical problems associated with this method, and these include:

1. The time taken to accurately draw the chart, and all the associated lines.
2. Reading the cut-off point from an imperfect drawing, rather than determining it mathematically.
3. One might wish to allow for some flexibility, and test other values for the parameters. On a hand-drawn chart, this is time-consuming and untidy, to the point of being impossible.

### Non-paper solutions

Hofstee produced a mathematical solution, ^7^ but it requires sorting and frequency pre-calculation and data inspection, and the mathematics involved is not rudimentary (requiring seven steps). Van Der Vleuten developed a useful one for SPSS, ^8^ but it uses expensive licensed software.

An Excel template designed by one of the authors (KM) already exists, and plotting the chart on Excel is certainly an improvement over the hand-drawn chart. However, it still requires the data to be pre-sorted and also requires the generation of the cumulative data. In addition, although the chart is drawn more accurately than by hand, it still requires a manual reading of the intersection point.

### An app

A search in both the Apple and Android app stores (conducted in January 2020 and again in March 2020) confirmed that there was no such app in either of the stores. To meet this need for a simple and accurate method of determining the Hofstee cut-off, we designed and developed a simple Android app. The app automatically sorts the data, draws the chart, and calculates the cut-off point algebraically. The result is a process that is faster and more accurate than the other mentioned methods.

For usability and evaluation, the app was designed according to the relevant principles laid out in the Mobile App Rating Scale (MARS). ^9^ The overall MARS scale is broad, and so does have a few weaknesses when applied to this type of app (e.g. it rates the entertainment value of the app), but it is still a useful guide. In addition, the app is available free of charge, and with no advertisements.

## Methods

### Implementation

The app, HofsteeCalc *,* was developed using MIT App Inventor Version 2 (builds nb182 through to nb186a). MIT App Inventor uses its own visual, block-based programming interface to develop Android and iOS apps. In addition to the internal code, the app uses three external sets of libraries and routines for browsing to and selecting the data file, ^10^ sorting the data, ^11^ and charting the data. ^12^ No user or device information is collected. The app is optimised for Android 2.1 and higher, API level 28, and requires permission to read from and store data to the device.

### Operation

**Figure 2.**
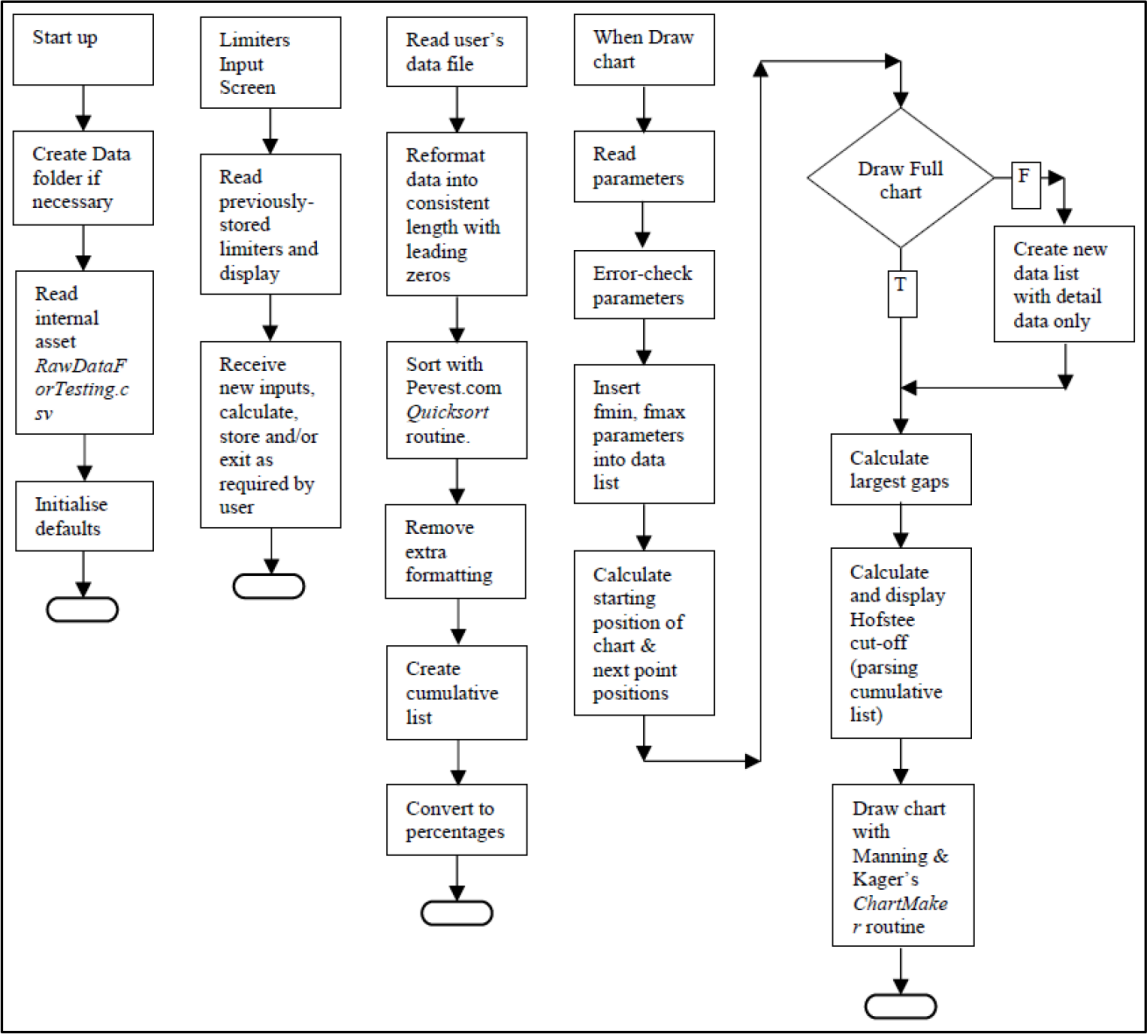
App workflow.

See Figure 2 for workflow chart.

The app automatically creates a data folder and has a test file that the user can use for testing before they insert their data.

**Figure 3.**
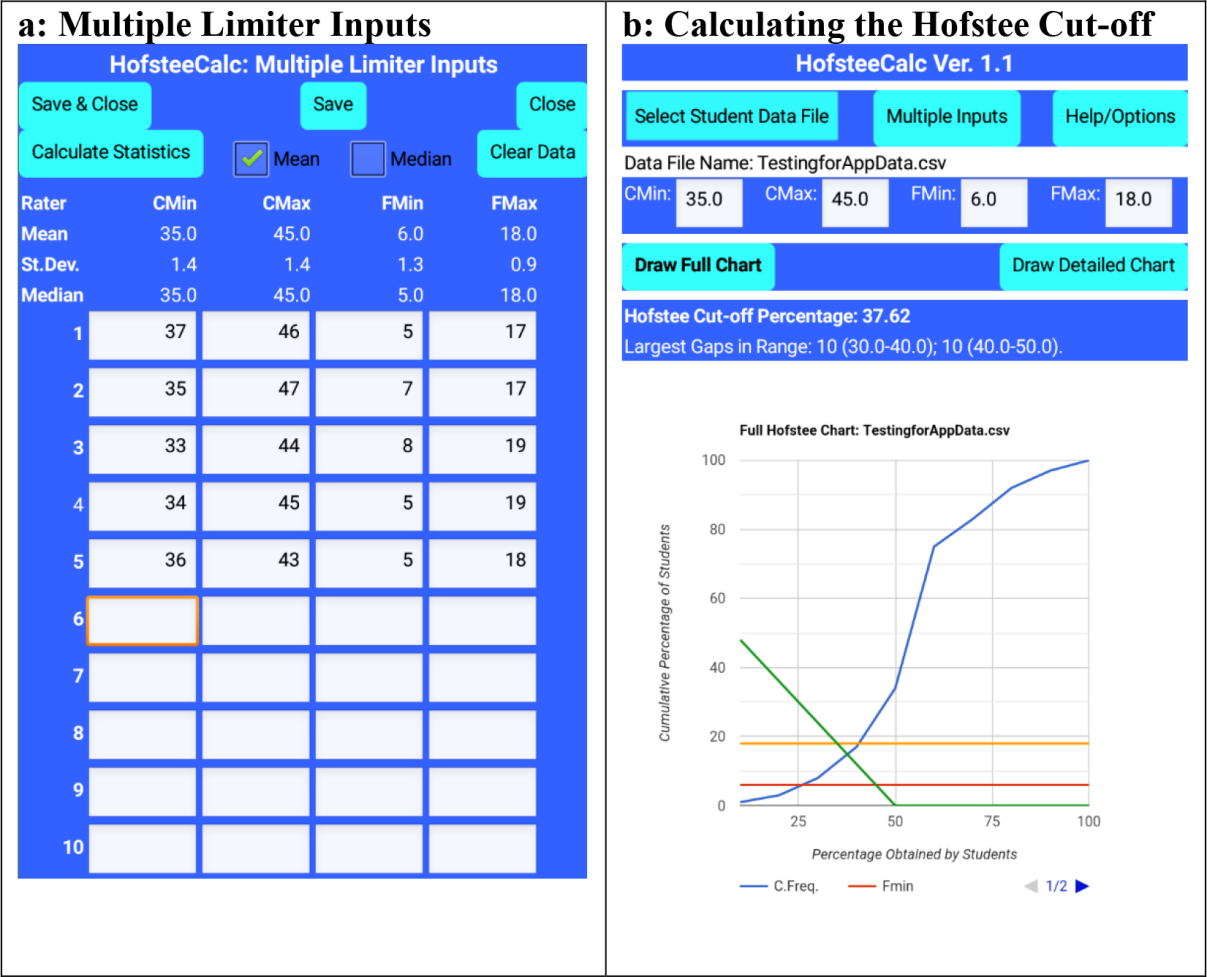
HofsteeCalc app: multiple judges’ parameters and output from Figure 1 data, where c _min_ is minimum cut-off score, c _max_ is maximum cut-off score, f _min_ is the lowest percentage of students that the judge feels should fail, and f _max_ is the highest percentage of students that the judge feels should fail.

The app allows each judge’s individual parameters to be entered (up to a maximum of 10 judges), and then calculates the means, standard deviations, and medians ( Figure 3a). The parameters are automatically stored if required and are available the next times the app runs. Alternately, the final means or medians can be entered directly into the main screen ( Figure 3b).

For data input, the student data need to be in a single-column standard.csv file. If the.csv file contains more than one column of data, only the first column will be read. The app automatically sorts the data, so these do not have to be pre-sorted by the user.

When the charts are to be drawn, the user can view either the chart of the whole data set (see Figure 3b: Draw Full Chart), or a detailed section (covering data which is within and close to the range of the parameters (see Figure 3b: Draw Detailed Chart). With pinching, users can zoom in and out of the charts.

As the focus of the app is a functional tool, it has a simple user interface, and includes a ‘Help’ screen that explains in detail how it is to be used. Although the app assumes a knowledge of the Hofstee method, it supplies additional references for the user. Allowing for personal preferences, it permits the user to change some user-interface colours to suit individual needs.

An ‘Options’ screen allows changing of the user-interface colour scheme of buttons, to suit the individual user’s needs.

### Central algorithm to algebraically determine the Hofstee cut-off

In the Hofstee chart, we know the x _1_y _1_ and x _2_y _2_ coordinates of line *AB* ( Figure 1). However, because the cumulative score line does not have an algebraic formula, calculating the intersection between this straight line and the cumulative line is not possible (using ‘best fit’ or ‘nearest neighbour’ might be possible but will not give 100% accuracy). It is for this reason that current users of the Hofstee method read the point manually from hand-drawn charts.

The data, however, are x _1_y _1_ and x _2_y _2_ coordinates of straight lines, and these coordinates are stored in an array (or list). So, the algebraic algorithm for determining the cut-off can be expressed in the following pseudo-code:

*For each straight line in the array of lines forming the cumulative line*

*Read the x _1_y _1_ and x _2_y _2_ coordinates of that line*

*Algebraically determine the intersection point (x _i_) of this straight line and line AB*

*IF x _1_ ≤ x _i_ ≤ x _2_ [there is no need to test the y coordinate]*

*THEN x _i_ is the cut-off point*

(If the cut-off ( *x _i_*) is a data point, then two lines would meet this condition, but that is no matter, as the point is identical.)

Readers may recognise that, because the cut-off point is determined algebraically, there is no need to draw the chart for the calculation. The chart, however, has been included in the app because most users are used to it, and also because they may wish to make manual adjustments to the parameters based on the visual reading of the data.

### App completion

After various early test versions, Version 1.0 of the app was completed in February 2021, and uploaded into the Google Play Store at: https://play.google.com/store/apps/details?id=appinventor.ai_itmeded.HofsteeCalc. Since then, small updates have been performed, and the app is currently on Ver. 1.1.

### App description and functionality

Conforming to the requirements laid out in the Introduction above, the app is available free of charge, with no advertisements. It does not require access to the internet, and it does not collect, store, or transmit any personal information about the user or the device.

### Alpha testing

The app was alpha tested on various real and hypothetical, sorted and unsorted datasets (see *Underlying data* ^13^), with up to 1,000 items, and consistently returned accurate results. For example, for the dataset used in Figure 1, the app calculated the cut-off at 37.62%, rather than “slightly less than 38%” (See Figure 3b).

The time to draw the chart and determine the cut-off from a dataset of unsorted, 1,000 randomly-generated numbers (MS-Excel 2019 RANDBETWEEN(1,100)), was approximately 2 seconds (Samsung S8, Model SM-G955FD, Android Ver. 9, Build PPR1.180610.011.G955FXXS6DTA1).

### Mobile App Rating Scale (MARS)

Using the Mobile App Rating Scale (MARS), ^9^ both authors independently measured the app against the scale, and arrived at a score of 4.07 and 3.88, respectively. As detailed above, this less-than-ideal score was expected, as the MARS includes items not entirely appropriate to such an app.

## Use case

For use cases, anonymised data sets are available in *Underlying data*. ^13^

An example of a use case utilised the data in the sheet HofsteeCalcRealDataClass01.csv.

The data set has 181 items, and the item values range from 43 to 97. The data set is unsorted.

The input parameters were determined as shown in Table 1.

**Table 1.** Use case input parameters for HofsteeCalcRealDataClass01.csv, where c _min_ is minimum cut-off score, c _max_ is maximum cut-off score, f _min_ is the lowest percentage of students that the judge feels should fail, and f _max_ is the highest percentage of students that the judge feels should fail.

Rater	c _min_	c _max_	f _min_	f _max_
1	50	60	1	4
2	44	56	3	8
3	41	52	4	8
4	45	54	5	8
5	45	53	2	7

**Figure 4.**
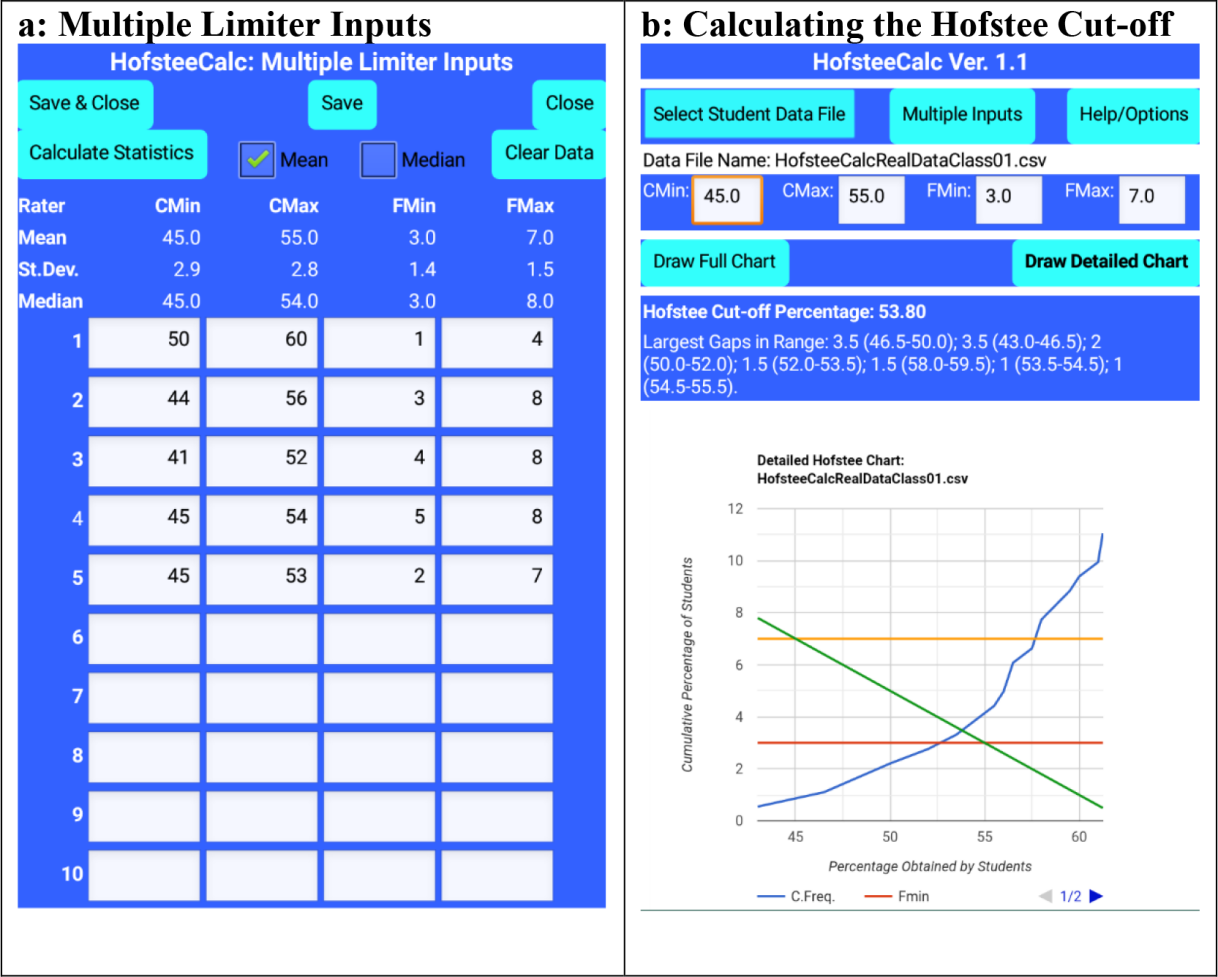
HofsteeCalc app: multiple judges’ parameters and output from HofsteeCalcRealDataClass01.csv, where c _min_ is minimum cut-off score, c _max_ is maximum cut-off score, f _min_ is the lowest percentage of students that the judge feels should fail, and f _max_ is the highest percentage of students that the judge feels should fail.

Based on this use case, Figure 4a shows the input parameters. Figure 4b shows the resultant ‘Detailed chart’, the Hofstee cut-off percentage (53.80), and the largest data gaps in the vicinity of the Hofstee cut-off percentage.

## Comments

This paper has described the successful design and development of a free, advertisement-free, Android app to calculate the Hofstee cut-off. The app meets basic design principles as established in the MARS scale, and alpha- and beta- testing has shown the app to be accurate and fast. The app is available in the Google Play app store (see *Software availability* ^14^).

Full usability and ease of use will be tested in the future through more rigorous, wide-spread testing among medical educators.

## Conclusions

When educating future health professionals, determining fair pass/fail cut-off points is crucial. The time taken to perform such procedures, however, adds to medical educators’ already over-burdened schedules, and competes with a range of other demands in this schedule, so it is inevitable that short-cuts and errors will occur. This research has traced the design and development of a tool that can both save time and improve accuracy when determining the Hofstee cut-off.

## Data availability

### Underlying data

Zenodo: HofsteeCalcDataSets. https://doi.org/10.5281/zenodo.4699233. ^13^

This project contains the following underlying data:

• RawDataForTesting.csv (data set that is built into the app’s assets).
• TestingForAppData.csv (data set used to generate Figure 1 and Figure 3b).
• HofsteeCalcRealDataClass01.csv (data set available for testing).
• HofsteeCalcRealDataClass02.csv (data set available for testing).
• HofsteeCalcRealDataClass03.csv (data set available for testing).
• HofsteeCalcRealDataClass04.csv (data set available for testing).

Data are available under the terms of the Creative Commons Attribution 4.0 International licenses (CC-BY 4.0).

## Software availability

Software available from Google Play app store: https://play.google.com/store/apps/details?id=appinventor.ai_itmeded.HofsteeCalc

Archived source code at time of publication: https://doi.org/10.5281/zenodo.4633140. ^14^

Licence: Creative Commons Attribution 4.0 International license (CC-BY 4.0).

## References

[1] Schauber SK Hecht M : How sure can we be that a student really failed? On the measurement precision of individual pass-fail decisions from the perspective of Item Response Theory. *Med Teach.* 2020 Dec 1;42(12):1374–84. 10.1080/0142159X.2020.1811844 32857621

[2] Norcini JJ : Standard setting on educational tests. *Med Educ.* 2003;37(5):464–9. 10.1046/j.1365-2923.2003.01495.x 12709190

[3] Downing SM Tekian A Yudkowsky R : Procedures for establishing defensible absolute passing scores on performance examinations in health professions education. *Teach Learn Med.* 2006;18(1):50–7. 10.1207/s15328015tlm1801_11 16354141

[4] Bandaranayake RC : Setting and maintaining standards in multiple choice examinations: AMEE Guide No. 37. *Med Teach.* 2008;30(9–10):836–45. 10.1080/01421590802402247 19117221

[5] Hofstee WKB : The case for compromise in educational selection and grading. In: Anderson SB Helmick JS , editors. *On Educational Testing* . San Francisco: Jossey-Bass;1983. p.109–27.

[6] Wyse AE Babcock B : An investigation of undefined cut scores with the Hofstee Standard-Setting Method. *Educ Meas Issues Pract.* 2017;36(4):28–34. 10.1111/emip.12163

[7] Hofstee W : Cesuurprobleem opgelost [The standard setting problem resolved]. *Onderz Van Onderwijs.* 1977;6:6–7.

[8] Van Der Vleuten C : Setting and maintaining standards in multiple choice examinations (AMEE Supplement 37.1). *Med Teach.* 2010;32:174–6. 10.3109/01421590903505703 20163238

[9] Stoyanov SR Hides L Kavanagh DJ : Mobile App Rating Scale: A New Tool for Assessing the Quality of Health Mobile Apps. *JMIR MHealth UHealth.* 2015;3(1):e27. 10.2196/mhealth.3422 25760773 PMC4376132

[10] Pura Vida Apps: File Extension [Internet]. *App Inventor Extensions* .2019 [cited 2020 Mar 1]. Reference Source

[11] Pevest.com: QuickSort routine for your App Inventor Apps! [Internet]. 2017 [cited 2020 Mar 1]. Reference Source

[12] Manning K Kager E : ChartMaker [Internet]. 2017 [cited 2020 Mar 1]. Reference Source

[13] Masters K : HofsteeCalcDataSets (Version Ver 1.1) [Data set]. *Zenodo* .2021. 10.5281/zenodo.4699233

[14] Masters K : HofsteeCalc (Version 1.1). *Zenodo* .2021, March 24. 10.5281/zenodo.4633140

