# Supplementary material for: Designing and developing an app to perform Hofstee cut-off calculations
Source: F1000Res. 2021 Oct 5;10:450. Originally published 2021 Jun 7. [Version 2] doi: 10.12688/f1000research.53096.2 (PMC8561612; doi:10.12688/f1000research.53096.2)
Supplement: Peer review history

# Peer review: Reviewer response for version 2

**DOI:** 10.5256/f1000research.77792.r96239

**Published:** 2021-11-01

**Authors:** Adam E. Wyse

**Affiliations:** 1 Renaissance, Arden Hills, MN, USA

I don’t have any additional feedback or suggestions for the revised manuscript.

Are the conclusions about the tool and its performance adequately supported by the findings presented in the article?

Partly

Is the rationale for developing the new software tool clearly explained?

Yes

Is the description of the software tool technically sound?

Partly

Are sufficient details of the code, methods and analysis (if applicable) provided to allow replication of the software development and its use by others?

Partly

Is sufficient information provided to allow interpretation of the expected output datasets and any results generated using the tool?

Yes

Reviewer Expertise:

Psychometrics; Standard Setting; Measurement; Item Response Theory; Assessment

I confirm that I have read this submission and believe that I have an appropriate level of expertise to confirm that it is of an acceptable scientific standard.

# Peer review: Reviewer response for version 2

**DOI:** 10.5256/f1000research.77792.r96240

**Published:** 2021-10-07

**Authors:** Benedict Canny

**Affiliations:** 1 Adelaide Medical School, University of Adelaide, Adelaide, SA, Australia

The authors have addressed the concerns outlined in the original review.

Are the conclusions about the tool and its performance adequately supported by the findings presented in the article?

Partly

Is the rationale for developing the new software tool clearly explained?

Yes

Is the description of the software tool technically sound?

Yes

Are sufficient details of the code, methods and analysis (if applicable) provided to allow replication of the software development and its use by others?

Yes

Is sufficient information provided to allow interpretation of the expected output datasets and any results generated using the tool?

Yes

Reviewer Expertise:

Education Research

I confirm that I have read this submission and believe that I have an appropriate level of expertise to confirm that it is of an acceptable scientific standard.

# Response

**Published:** 2021-10-07

**Authors:** Ken Masters

**Affiliations:** Sultan Qaboos University, Oman

Thank you very much for taking the time to review our paper, and re-reading our 2nd version.  Much appreciated.

# Peer review: Reviewer response for version 1

**DOI:** 10.5256/f1000research.56446.r90318

**Published:** 2021-08-11

**Authors:** Benedict Canny

**Affiliations:** 1 Adelaide Medical School, University of Adelaide, Adelaide, SA, Australia

This paper describes the development and use of the an app to help with the use of the Hofstee method. I suspect it will be of considerable use to those who adopt this method. Importantly, the app only addresses the analysis of data, once the appropriate data have been generated, and users should fully acquaint themselves with strengths and weakness of the Hofstee standard setting method prior to use. Indeed, this advice could be applied to all standard setting approaches, as all have their pluses and minuses.

 While aware of the Hofstee method, I have never used in practice, so I am unable to comment on the practicalities of this method when compared with performing this task long hand.

 I am concerned about some of the terminology used in the paper. The authors use the words to "accurate" and "accuracy" in the Alpha Testing and Conclusions sections of the manuscript, and report cut scores to two significant decimal figures. I suspect that they mean the term "precision", as accuracy is a measure of the proximity of the estimated value to the "true" value, and would be best determined by comparing this method to another (e.g. van der Vleuten's method referred to in the paper), or a "gold" standard (making calculations long hand). The authors have not reported on the effect of the app on the "error rate" of using the method, and these additional data would be useful, if available.

 Finally, the point is made in the conclusion that "determining fair pass/fail cut-off points is crucial". This method will only increase fairness if the error rate is reduced, and not the inherent "fairness" of Hofstee method, nor, indeed, any other standard setting method.

 In conclusion, I suspect this app will be useful for those who use the Hofstee method. It would be nice to see an iOS version.

Are the conclusions about the tool and its performance adequately supported by the findings presented in the article?

Partly

Is the rationale for developing the new software tool clearly explained?

Yes

Is the description of the software tool technically sound?

Yes

Are sufficient details of the code, methods and analysis (if applicable) provided to allow replication of the software development and its use by others?

Yes

Is sufficient information provided to allow interpretation of the expected output datasets and any results generated using the tool?

Yes

Reviewer Expertise:

Education Research

I confirm that I have read this submission and believe that I have an appropriate level of expertise to confirm that it is of an acceptable scientific standard, however I have significant reservations, as outlined above.

# Response

**Published:** 2021-09-05

**Authors:** Ken Masters

**Affiliations:** Sultan Qaboos University, Oman

We thank you for your comments. We shall take them into account and address them in more detail when we submit Version 2 of the paper.

# Response

**Published:** 2021-09-22

**Authors:** Ken Masters

**Affiliations:** Sultan Qaboos University, Oman

We have now had the opportunity to respond in detail to this review. Below, we indicate the Reviewer’s comments and our response.

**Reviewer’s Comment

 Approved With Reservations

**Authors’ Response

 We thank the reviewer for their comments, and trust that our responses below and the changes to Version 1 of the paper will be to their satisfaction.

**Reviewer’s Comment

 This paper describes the development and use of the an app to help with the use of the Hofstee method. I suspect it will be of considerable use to those who adopt this method. Importantly, the app only addresses the analysis of data, once the appropriate data have been generated, and users should fully acquaint themselves with strengths and weakness of the Hofstee standard setting method prior to use. Indeed, this advice could be applied to all standard setting approaches, as all have their pluses and minuses.

 While aware of the Hofstee method, I have never used in practice, so I am unable to comment on the practicalities of this method when compared with performing this task long hand.

 I am concerned about some of the terminology used in the paper. The authors use the words to "accurate" and "accuracy" in the Alpha Testing and Conclusions sections of the manuscript, and report cut scores to two significant decimal figures. I suspect that they mean the term "precision", as accuracy is a measure of the proximity of the estimated value to the "true" value, and would be best determined by comparing this method to another (e.g. van der Vleuten's method referred to in the paper), or a "gold" standard (making calculations long hand). The authors have not reported on the effect of the app on the "error rate" of using the method, and these additional data would be useful, if available.

**Authors’ Response

 Thank you for this comment. Given that the final accurate answer is best determined by finding the intersect point of the two relevant lines in the chart, the final accurate result can be independently calculated with those known coordinates. (The main function of the app is to determine those two lines). So, for further verification, we have tested these coordinates using an online calculator (AMBrSoft, http://www.ambrsoft.com/MathCalc/Line/TwoLinesIntersection/TwoLinesIntersection.htm), and the app’s results are shown to be accurate to two decimal places. We also confirmed this with a enlarged hand-drawn calculation. Version 2 of the paper has been amended to reflect this, and the relevant coordinates have been placed in the paper, so that users can test these results independently.

 The reviewer is correct, though, that this level of increased accuracy is only against the methods that require manual reading of the charts, so, we feel we can now claim that the app is more accurate than the standard manual process, which could not have the same level of accuracy, and we have amended the paper to reflect this.

**Reviewer’s Comment

 Finally, the point is made in the conclusion that "determining fair pass/fail cut-off points is crucial". This method will only increase fairness if the error rate is reduced, and not the inherent "fairness" of Hofstee method, nor, indeed, any other standard setting method.

**Authors’ Response

 Yes, we agree. Given that the accuracy of the app has been confirmed (and can easily be verified by readers), we feel that the general statement that “determining fair pass/fail cut-off points is crucial” is valid.

**Reviewer’s Comment

 In conclusion, I suspect this app will be useful for those who use the Hofstee method. It would be nice to see an iOS version.

**Authors’ Response

 Yes, it would. Although an iOS app is planned, it would be premature to develop the app (or even allude to it in the paper) until the Android version has been accepted by the academic community.

- Is the rationale for developing the new software tool clearly explained?

Yes

- Is the description of the software tool technically sound?

Yes

- Are sufficient details of the code, methods and analysis (if applicable) provided to allow replication of the software development and its use by others?

Yes

- Is sufficient information provided to allow interpretation of the expected output datasets and any results generated using the tool?

Yes

- Are the conclusions about the tool and its performance adequately supported by the findings presented in the article?

Partly

**Authors’ Response

 We trust that our explanation and changes to the paper are to the reviewer’s satisfaction.

# Peer review: Reviewer response for version 1

**DOI:** 10.5256/f1000research.56446.r86927

**Published:** 2021-07-14

**Authors:** Adam E. Wyse

**Affiliations:** 1 Renaissance, Arden Hills, MN, USA

I appreciate the opportunity to review the article “Designing and developing an app to perform Hofstee cut-off calculations” by Ken Masters and Nadia Al-Wardy. I support the idea of developing simple software tools that people can use to perform standard setting. This is an area of definite need, especially in medical education contexts. I am unaware of a widely available software application to perform the Hofstee method. In most cases that I have seen the Hofstee method used, the people leading the standard setting use Excel or another statistical software package, such as R, to perform the calculations and figure out the cut score. In this sense, the app offered by Masters and Al-Wardy may be beneficial to people who want to perform the Hofstee method and do not have already developed software to perform the Hofstee method. I also liked how the authors provided screen shots of how the app works and several figures and examples throughout the article.

 I do have several suggestions for changes to the article and app. First, the authors suggest in the section Step 1: Evaluation of Judges that “We should further note that judges will generally use the Angoff or similar method to determine these.” This statement is not completely accurate. Wyse (2020) ^1^ discusses several different methods for performing the Hofstee method. One strategy is to figure out the minimum and maximum cut scores from the panel of judges using the Angoff (1971) ^2^ method or another test-centered method, such as the Bookmark method (Lewis, Mitzel, & Green, 1996) ^3^. However, this method is not the most common strategy I have seen used to collect these data. It is more common to directly ask panellists to answer four open-ended questions to solicit the data needed to estimate the Hofstee cut score. In addition, it should be noted that even if the Angoff method is used with the Hofstee method that the data on the lowest and high percentages of students that a judge feels should fail the test (which is sometimes alternatively phrased in terms of the highest and lowest students that should pass the test) need to be directly collected from individual judges.

 It should also be clear that the description of using the Angoff method to provide data to calculate the cut scores appears to be inconsistent with how the app works in Figure 3. In Figure 3, the authors show a screen with input for each rater. It is not possible to use the Angoff method to provide the multiple limiter input data shown on this screen. The app could be improved if it allowed for data from an Angoff standard setting or other test-centered method to be input. This input could be either entering the minimum and maximum cut scores from a test-centered standard setting method or each judge’s cut score from such a standard setting. It would also be beneficial if the app allowed for an option to input the lowest and highest passing rates and a corresponding graph instead of failure rates. I have commonly seen the method used with passing rates instead of failure rates. Judges sometimes find it easier to conceptualize and use pass rates as many credentialing and licensing organizations as well as accrediting bodies use passing rates instead of failure rates. Finally, it appears that the app requires that cut scores needs to be expressed as a percentage correct. It would be useful if the app also allowed for raw scores to be input as an option as I have seen raw scores used in many different standard settings.

 Another area for potential improvement is the example shown in Figure 1. The example shown in Figure 1 appears to be based on data from a single judge. While it is possible to determine the Hofstee cut score for each individual judge, this is rarely done as the authors note in Step 2: Determining the arithmetic mean. The figure would be more beneficial if it focused on data from a group of judges as this is how the method is typically implemented.

 There is a fourth practical and very real problem that occurs with the Hofstee method that is not described by the authors. Wyse and Babcock (2017) ^4^ illustrate that the Hofstee method can produce undefined cut scores where the Hofstee line segment does not intersect with the failure rate or pass rate curve. This problem is a more serious issue than the three issues described by the authors as it implies that a cut score cannot be estimated. This issue should be mentioned in this section. Wyse and Babcock (2017) ^4^ offer a solution for how to simply solve this issue, which involves extending the Hofsee line segment so that it intersects with the pass rate or failure rate curve. The other three practical issues described by the authors are easy to solve with Excel or other statistical software for technical savvy standard setters. 

 It should also be pointed out that the way the authors describe calculating the Hofstee cut score is different than the way that I typically think about doing it. The authors description of their method based on arrays is not easy to understand and follow, especially for many educators who may use the app. Wyse and Babcock (2017) ^4^ offer an easy way to determine the cut scores for the Hofstee method that guarantees a solution even if the Hofstee line segment does not intersect the pass rate or failure rate curve. The authors should consider implementing this method in their app and provide a corresponding description in the article. The strategy involves finding the equation for the line that passes through the two points represented by the means of the data collected from the standard-setting judges that was described in earlier section of the article by the authors. Then, one inputs range of possible scores on the exam to figure out the estimated pass rate or failure rate (depending on whether pass rate or failure rate data are collected from judges) for every possible score on the exam. The last step is to compare the estimated pass rates or failure rates to the observed pass rates or failure rates on the exam. The score with the smallest absolute difference between the observed and estimated pass rates or failure rates is the cut score.

 In summary, I think having a simple software app to perform Hofstee calculations is useful. However, the current version of the app does not cover all possible ways that the Hofstee method may be implemented which may limit its utility. If the authors made several changes based on the suggestions in this review, I think the app and article would have more utility and be easier to make sense for users.

Are the conclusions about the tool and its performance adequately supported by the findings presented in the article?

Partly

Is the rationale for developing the new software tool clearly explained?

Yes

Is the description of the software tool technically sound?

Partly

Are sufficient details of the code, methods and analysis (if applicable) provided to allow replication of the software development and its use by others?

Partly

Is sufficient information provided to allow interpretation of the expected output datasets and any results generated using the tool?

Yes

Reviewer Expertise:

Psychometrics; Standard Setting; Measurement; Item Response Theory; Assessment

I confirm that I have read this submission and believe that I have an appropriate level of expertise to confirm that it is of an acceptable scientific standard, however I have significant reservations, as outlined above.

## References

[1] : Comparing Cut Scores from the Angoff Method and Two Variations of the Hofstee and Beuk Methods. *Applied Measurement in Education* .2020;33(2) : 10.1080/08957347.2020.1732385 159-173 10.1080/08957347.2020.1732385

[2] : Scales, norms, and equivalent scores. In R. L. Thorndike (Ed.), Educational measurement (2nd ed.). *American Council on Education* .1971;508-597

[3] : Standard setting: A Bookmark approach. In D. R. Green (Chair), IRT-based standard setting procedures utilizing behavioral anchoring. *Symposium presented at the Council of Chief State School Officers National Conference on Large-Scale Assessment* .1996;

[4] : An Investigation of Undefined Cut Scores With the Hofstee Standard-Setting Method. *Educational Measurement: Issues and Practice* .2017;36(4) : 10.1111/emip.12163 28-34 10.1111/emip.12163

# Response

**Published:** 2021-07-19

**Authors:** Ken Masters

**Affiliations:** Sultan Qaboos University, Oman

We thank you for your detailed comments. We shall take them into account and address them in more detail when we have received responses from other reviewers.

# Response

**Published:** 2021-09-22

**Authors:** Ken Masters

**Affiliations:** Sultan Qaboos University, Oman

We have now had the opportunity to respond in detail to this review. Below, we indicate the Reviewer’s comments and our response.

**Reviewer’s Comment

 Approved With Reservations

**Authors’ Response

 We thank the reviewer for their very detailed comments, and trust that our responses below and the changes to Version 1 of the paper will be to their satisfaction.

**Reviewer’s Comment

 I appreciate the opportunity to review the article “Designing and developing an app to perform Hofstee cut-off calculations” by Ken Masters and Nadia Al-Wardy. I support the idea of developing simple software tools that people can use to perform standard setting. This is an area of definite need, especially in medical education contexts. I am unaware of a widely available software application to perform the Hofstee method. In most cases that I have seen the Hofstee method used, the people leading the standard setting use Excel or another statistical software package, such as R, to perform the calculations and figure out the cut score. In this sense, the app offered by Masters and Al-Wardy may be beneficial to people who want to perform the Hofstee method and do not have already developed software to perform the Hofstee method. I also liked how the authors provided screen shots of how the app works and several figures and examples throughout the article.

**Authors’ Response

 We thank the reviewer for their overall frank comments.

**Reviewer’s Comment

 I do have several suggestions for changes to the article and app. First, the authors suggest in the section Step 1: Evaluation of Judges that “We should further note that judges will generally use the Angoff or similar method to determine these.” This statement is not completely accurate. Wyse (2020) ^1^ discusses several different methods for performing the Hofstee method. One strategy is to figure out the minimum and maximum cut scores from the panel of judges using the Angoff (1971) ^2^ method or another test-centered method, such as the Bookmark method (Lewis, Mitzel, & Green, 1996) ^3^ . However, this method is not the most common strategy I have seen used to collect these data. It is more common to directly ask panellists to answer four open-ended questions to solicit the data needed to estimate the Hofstee cut score. In addition, it should be noted that even if the Angoff method is used with the Hofstee method that the data on the lowest and high percentages of students that a judge feels should fail the test (which is sometimes alternatively phrased in terms of the highest and lowest students that should pass the test) need to be directly collected from individual judges.

**Authors’ Response

 Thank you for this comment. The published texts that we were using (Bandaranayake 2008; Wyse and Babcock 2017) indicated that the Hofstee was frequently used in conjunction with the Angoff method and so we mentioned that. As this is not central to the paper, however, the sentence has been deleted.

**Reviewer’s Comment

 It should also be clear that the description of using the Angoff method to provide data to calculate the cut scores appears to be inconsistent with how the app works in Figure 3. In Figure 3, the authors show a screen with input for each rater. It is not possible to use the Angoff method to provide the multiple limiter input data shown on this screen. The app could be improved if it allowed for data from an Angoff standard setting or other test-centered method to be input. This input could be either entering the minimum and maximum cut scores from a test-centered standard setting method or each judge’s cut score from such a standard setting. It would also be beneficial if the app allowed for an option to input the lowest and highest passing rates and a corresponding graph instead of failure rates. I have commonly seen the method used with passing rates instead of failure rates. Judges sometimes find it easier to conceptualize and use pass rates as many credentialing and licensing organizations as well as accrediting bodies use passing rates instead of failure rates.

**Authors’ Response

 Thank you for this comment. We apologise for the misunderstanding about what is being portrayed in Figure 3, and we acknowledge that the misunderstanding is because the caption in Figure 1 (raised by the Reviewer below), and then the paragraph that immediately precedes Figure 3, which gives the impression that a single judge’s information has been input (in Figure 3b).

 As a result, in addition to the corrections to the Figure 1 caption, we have altered the description in the paragraph preceding Figure 3 in order to clarify the process.

**Reviewer’s Comment

 Finally, it appears that the app requires that cut scores needs to be expressed as a percentage correct. It would be useful if the app also allowed for raw scores to be input as an option as I have seen raw scores used in many different standard settings.

**Authors’ Response

 Thank you for this comment. This may be an addition to Version 2 of the app, but the current literature (e.g. Bandaranayake 2008; Burr *et al* 2016) indicates percentage scores as the input, and so, it is prudent for Version 1 of the app to stick to the more common process as described in the dominant literature.

**Reviewer’s Comment

 Another area for potential improvement is the example shown in Figure 1. The example shown in Figure 1 appears to be based on data from a single judge. While it is possible to determine the Hofstee cut score for each individual judge, this is rarely done as the authors note in Step 2: Determining the arithmetic mean. The figure would be more beneficial if it focused on data from a group of judges as this is how the method is typically implemented.

**Authors’ Response

 Thank you for this point. Figure 1 is an illustrative example showing the chart after the arithmetic means of the parameters have been determined. As mentioned above, however, we acknowledge that the caption was erroneous, and it (and the sentence preceding it) have now been corrected. 

**Reviewer’s Comment

 There is a fourth practical and very real problem that occurs with the Hofstee method that is not described by the authors. Wyse and Babcock (2017) ^4^ illustrate that the Hofstee method can produce undefined cut scores where the Hofstee line segment does not intersect with the failure rate or pass rate curve. This problem is a more serious issue than the three issues described by the authors as it implies that a cut score cannot be estimated. This issue should be mentioned in this section. Wyse and Babcock (2017) ^4^ offer a solution for how to simply solve this issue, which involves extending the Hofsee line segment so that it intersects with the pass rate or failure rate curve. The other three practical issues described by the authors are easy to solve with Excel or other statistical software for technical savvy standard setters. 

**Authors’ Response

 We are reluctant to get into a discussion about the weaknesses of the Hofstee method or how to solve problems associated with it. To do so, and to do such a discussion justice (and view all arguments equally from all sides), is a paper by itself. In our discussion of the Hofstee Method, the aim is simply to explain it so that the functioning of the app is understood. Nevertheless, as readers may be expecting a discussion of the strengths and weaknesses of the Hofstee Method, we have inserted the sentence: “While there are weaknesses with the method, and they have been discussed elsewhere, this paper is focused on describing the method, and then describing an app that applies the method.” And the “elsewhere” in the sentence cites the Wyse & Babcock article mentioned by the reviewer.

**Reviewer’s Comment

 It should also be pointed out that the way the authors describe calculating the Hofstee cut score is different than the way that I typically think about doing it. The authors description of their method based on arrays is not easy to understand and follow, especially for many educators who may use the app. Wyse and Babcock (2017) ^4^ offer an easy way to determine the cut scores for the Hofstee method that guarantees a solution even if the Hofstee line segment does not intersect the pass rate or failure rate curve. The authors should consider implementing this method in their app and provide a corresponding description in the article. The strategy involves finding the equation for the line that passes through the two points represented by the means of the data collected from the standard-setting judges that was described in earlier section of the article by the authors. Then, one inputs range of possible scores on the exam to figure out the estimated pass rate or failure rate (depending on whether pass rate or failure rate data are collected from judges) for every possible score on the exam. The last step is to compare the estimated pass rates or failure rates to the observed pass rates or failure rates on the exam. The score with the smallest absolute difference between the observed and estimated pass rates or failure rates is the cut score.

**Authors’ Response

 Thank you for your suggestion. The proposal for modification by Wyse and Babcock (2017) is, indeed, interesting. There are also others (e.g. Burr *et al* 2016). 

 The app (and, therefore, the article), however, is designed to determine the Hofstee cut-off as it is commonly practiced (e.g. Bandaranayake 2008). All proposed modifications cannot be implemented, and, if the app were to favour one modification over another, we would be open to criticisms of favouritism and of promoting and endorsing one modification over others. In this instance, the criticisms would be particularly sharp, because this modification has been proposed by one of the modification authors, who is also a reviewer of this paper. If we were to implement this modification (and not others), we would lay ourselves open to the accusation that we had made the modification to the app and the paper primarily to gain favour from the reviewer in the hopes of a more favourable review. As the reviewer can appreciate, both the authors and the journal would then be under pressure to retract such a paper (and remove the app from the app store).

 That said, we could certainly envision a future version of the app that, based on a detailed literature review of all possible Hofstee variations and modification, would attempt to implement them all, allowing users to choose their favourite modification.

**Reviewer’s Comment

 In summary, I think having a simple software app to perform Hofstee calculations is useful. However, the current version of the app does not cover all possible ways that the Hofstee method may be implemented which may limit its utility. If the authors made several changes based on the suggestions in this review, I think the app and article would have more utility and be easier to make sense for users.

**Authors’ Response

 Thank you for your suggestion. We trust that our explanation and paper’s modifications detailed above satisfies the reviewer.

- Is the rationale for developing the new software tool clearly explained?

Yes

- Is the description of the software tool technically sound?

Partly

**Authors’ Response

 We trust that our explanation and changes to the paper are to the reviewer’s satisfaction.

- Are sufficient details of the code, methods and analysis (if applicable) provided to allow replication of the software development and its use by others?

Partly

**Authors’ Response

 We trust that our explanation and changes to the paper are to the reviewer’s satisfaction.

- Is sufficient information provided to allow interpretation of the expected output datasets and any results generated using the tool?

Yes

- Are the conclusions about the tool and its performance adequately supported by the findings presented in the article?

Partly

**Authors’ Response

 We trust that our explanation and changes to the paper are to the reviewer’s satisfaction.
